# Efficacy and safety of metformin during pregnancy: an update

**DOI:** 10.1007/s12020-023-03550-0

**Published:** 2023-10-05

**Authors:** Stavroula A. Paschou, Almog Shalit, Eleni Gerontiti, Kleoniki I. Athanasiadou, Theodoros Kalampokas, Theodora Psaltopoulou, Irene Lambrinoudaki, Eleni Anastasiou, Bruce H. R. Wolffenbuttel, Dimitrios G. Goulis

**Affiliations:** 1 Endocrine Unit and Diabetes Center, Department of Clinical Therapeutics, Alexandra Hospital, School of Medicine, National and Kapodistrian University of Athens, Athens, Greece; 2 Second Department of Obstetrics and Gynecology, Aretaieion University Hospital, School of Medicine, National and Kapodistrian University of Athens, Athens, Greece; 3 Department of Endocrinology, Alexandra Hospital, Athens, Greece; 4 Department of Endocrinology, University of Groningen, University Medical Center Groningen, Groningen, The Netherlands; 5 Unit of Reproductive Endocrinology, First Department of Obstetrics and Gynecology, School of Medicine, Aristotle University of Thessaloniki, Thessaloniki, Greece

**Keywords:** Gestational diabetes mellitus, GDM, Metformin, Pregnancy

## Abstract

During the last decades, gestational diabetes mellitus (GDM) prevalence has been on the rise. While insulin remains the gold standard treatment for GDM, metformin use during pregnancy is controversial. This review aimed to comprehensively assess the available data on the efficacy and safety of metformin during pregnancy, both for the mother and the offspring. Metformin has been validated for maternal efficacy and safety, achieving comparable glycemic control with insulin. Additionally, it reduces maternal weight gain and possibly the occurrence of hypertensive disorders. During the early neonatal period, metformin administration does not increase the risk of congenital anomalies or other major adverse effects, including lower APGAR score at 5 min, neonatal intensive care unit admissions, and respiratory distress syndrome. Several studies have demonstrated a reduction in neonatal hypoglycemia. Metformin has been associated with an increase in preterm births and lower birth weight, although this effect is controversial and depends on the indication for which it was administered. Evidence indicates possible altered fetal programming and predisposition to childhood obesity and metabolic syndrome during adulthood after use of metformin in pregnancy. With critical questions still requiring a final verdict, ongoing research on the field must be conducted.

## Introduction

Gestational hyperglycemia cases are exhibiting a staggering surge in recent years and health care providers have to confront and manage the associated health consequences. The adverse outcomes include spontaneous abortion, pre-eclampsia, fetal anomalies, fetal demise, macrosomia, cesarean section, neonatal hypoglycemia, and neonatal respiratory distress syndrome [1]. The 10^th^ edition of the International Diabetes Atlas states that hyperglycemia during pregnancy affects 16.7% of pregnancies worldwide, accounting for 21.1 million affected live births [2]. According to the International Diabetes Federation (IDF), hyperglycemia in pregnancy can be classified as gestational diabetes mellitus (GDM) (80.3%), pre-gestational diabetes (10.6%), and diabetes diagnosed during pregnancy (overt diabetes) (9.1%) [2].

Universal screening for GDM at 24–28 gestational weeks has been established and is performed with a one- or a two-step approach according to various diagnostic criteria, without universal consensus [1, 3–5]. According to the American Diabetes Association (ADA) guidelines, GDM diagnosis can be accomplished either with the one-step 75-g oral glucose tolerance test (OGTT) (IADPSG criteria) or with the two-step approach that consists of a 50 g screening test followed by a 3-hour 100-g OGTT for positive subjects (Carpenter-Coustan criteria) [6, 7].

Invariably, management involves lifestyle modifications, including nutritional intervention, physical exercise, and weight loss, if indicated [6]. If lifestyle changes are insufficient for adequate glycemic control, pharmaceutical intervention follows [1, 3, 6]. Although insulin is the gold standard treatment during pregnancy, the risk of hypoglycemia and weight gain, as well as the financial burden, maintenance requirements and patient education time, hinder patient compliance and impede treatment acceptance [1]. Currently, only the oral agents metformin and glyburide are acceptable alternatives, as the other hypoglycemic options lack long-term safety data [1]. The available data indicate patients’ preference for the oral administration route of metformin rather than insulin’s subcutaneous injection, which has resulted in enhanced compliance and satisfaction [8–10]. However, caution should be taken as both agents cross the placenta, unlike insulin [11].

Guidelines for the pharmaceutical management of hyperglycemia during pregnancy vary. ADA recommends insulin as the first-line agent and suggests metformin as a secondary option, if necessary [1]. In the guidelines of the Canadian Diabetes Association, insulin remains the gold standard, while metformin is considered as an alternative approach only in patients with GDM [4]. Comparably, a board of Italian scientific societies reported that metformin could be used for diabetes in pregnancy as a second-line treatment or in addition to insulin, to reduce its dose, especially in obese women [12]. According to two expert Greek teams, which were commissioned to construct the national guidelines, no antidiabetic drug, except insulin, should be recommended during pregnancy [13–15]. In contrast, the UK’s NICE guidelines recommend metformin as first-line treatment and insulin only under special conditions [3]. Notably, the Italian guidelines introduce metformin considerations for pregnant women with a body mass index (BMI) over 35 kg/m^2^ or women with polycystic ovary syndrome (PCOS), especially when BMI is over 30 kg/m^2^ [12]. However, ADA recommends that metformin should be discontinued by the end of the first trimester, when prescribed pre-pregnancy in women with PCOS to assist with conception [1]. Further indications, other than obesity or PCOS, are being investigated with limited evidence [16–18]. Potential factors contributing to these divergences in various national guidelines include different priorities, such as economical or medico-legal considerations.

Metformin is widely prescribed to pregnant women, but its safety has yet to be established. Although initial studies of metformin during the antenatal and immediate postnatal period found no negative effect [19–21], recent research has suggested potential effects on fetal metabolic programming and long-term consequences [22, 23].

This review aims to comprehensively assess the available data on the efficacy and safety of metformin during pregnancy for both mother and offspring. Table 1 provides a concice summary of key attributes pertaining to the randomised controlled trials (RCTs), that will be utilised during the review.

**Table 1:** Study characteristics of included RCTs

Study	Publication year	Indication	Intervention	Control	Initial Met dose (/day)	Max Met dose (/day)
Rowan et al. (MiG) [8]	2008	GDM	Met (±insulin)	Insulin	500 mg or 1000 mg	2500 mg
Niromanesh et al*.* [75]	2012	GDM	Met (±insulin)	Insulin	1000 mg	2500 mg
Spaulonci et al. [76]	2013	GDM	Met (±insulin)	Insulin	1700 mg	2550 mg
Tertti et al. [26]	2013	GDM	Met (±insulin)	Insulin	500 mg	2000 mg
Ainuddin et al. [10]	2015	GDM	Met (±insulin)	Insulin	500 mg	2500 mg
Rowan et al. (MiG TOFU) [22]	2018	GDM	Met (±insulin)	Insulin		
Eid et al. [27]	2018	GDM	Met	Insulin	500 mg	2500 mg
Dunne et al. (EMERGE) [35]	2022	GDM	Usual care + Met	Usual care + placebo	500 mg	2500 mg
Ainuddin et al. [9]	2015	T2DM	Met (±insulin)	Insulin	500 mg	2500 mg
Feig et al. (MiTy) [31]	2020	T2DM	Insulin + Met	Insulin + placebo	500 mg	2000 mg
Vanky et al. (PregMet) [43]	2010	PCOS	Met	Placebo	1000 mg	2000 mg
Løvvik et al. (PregMet2) [24]	2016	PCOS	Met	Placebo	1000 mg	2000 mg
Syngelaki et al. [17]	2016	Obesity	Met	Placebo	1000 mg	3000 mg
Dodd et al. (GRoW) [47]	2019	Overweight Obesity	Met	Placebo	500 mg	2000 mg
Valdés et al. [48]	2018	Pre-gestational insulin resistance	Met	Placebo	1700 mg	1700 mg
Cluver et al. [74]	2021	Pre-eclampsia	Met	Placebo	1500 mg	3000 mg

*Met* Metformin, *RCTs* randomized controlled trials, *T2DM* type 2 diabetes mellitus

## Maternal outcomes: metformin for gestational and type 2 diabetes

### Maternal weight gain

The available data consistently indicate reduced weight gain with metformin use during pregnancy compared to controls. This effect is observed regardless of treatment indication [type 2 diabetes mellitus (T2DM), GDM, PCOS] [8, 9, 20, 24–31]. The difference in weight gain ranges from 0.47 kg to 1.8 kg. Obesity significantly increases the risk of pregnancy complications related to metabolic diseases (Tables 1–3). Therefore, such complications can be reduced by minimal weight gain during pregnancy [29]. Notably, studies on the effect of metformin postpartum weight change are still inconclusive [32–34].

### Glycemic control

Numerous studies report that metformin is equivalent or even superior to insulin in glycemic control during pregnancy [8, 20, 25, 28, 29, 31]. Glycemic control can be assessed by various modalities, such as glycated hemoglobin (HbA_1c_), and fasting, postprandial, and random blood glucose. Notably, the threshold for target glucose concentrations is inconsistent between study protocols. Specifically, the maximum fasting glucose concentrations range from 5.5 to 6.1 mmol/L (100 to 110 mg/dL), and postprandial concentrations were measured at 1, 1.5 or 2 h with differing limits [8, 9, 26]. Clinical trials on the topic continue to arise. EMERGE trial is an ongoing phase III placebo-controlled clinical trial studying metformin’s early initiation effectiveness and safety on GDM [35]. Another ongoing initiative is the MATCh-GDM trial, which evaluates GDM pathogenetic mechanisms, to establish a more individualized treatment approach [36].

### Need for additional insulin

Insulin is added when glycemic targets are not reached during treatment with metformin. Depending on the study protocol, women in need of supplementary insulin were excluded from the analysis [27], continued in the metformin group [22], or examined as an additional group [9, 10]. In the Mig trials, 46.3% of women in the metformin group were administered add-on insulin. These patients had higher BMI and elevated glycemic concentrations at randomization compared to women who achieved their treatment goals with metformin alone [8]. Another RCT reported that the women receiving metformin and required additional insulin (20.9%) were older, had an earlier screening, and had higher fructosamine and HbA_1c_ concentrations at baseline. Further analysis demonstrated that fructosamine at baseline could predict the need for additional insulin [relative risk (RR) 4.6, 95% CI 1.6 to 13.6; *p* = 0.006] [26]. In a case-control study comparing metformin with metformin plus insulin in GDM patients, 22.94% of the cohort required supplementary insulin. Fasting glucose concentrations below 90 mg/dL at screening and primiparity were associated with reduced odds of requiring combination treatment [odds ratio (OR) 0.438, 95% confidence interval (CI) 0.235 to 0.815; *p* = 0.009 and OR 0.28, 95% CI 0.111 to 0.704; *p* = 0.007, respectively]. In contrast, obesity was correlated with an increased risk for metformin failure (OR 2.072, 95% CI 1.063 to 4.039; *p* = 0.032) [37]. Lastly, in an RCT of T2DM, 84.9% of women in the metformin group required additional insulin [9]. A possible explanation of disparities can be variations in genetic and phenotypic characteristics of studied populations [9]. Notably, in addition to inconsistencies in group formation, the dosing, titration, and initiation of treatment vary between studies. Thus, the establishment of universal therapeutic guidelines is necessary [8, 10, 26, 27].

### Pregnancy-related hypertensive disorders

Several studies support a trend for reduced risk of gestational hypertensive disorders with metformin use [9, 25, 28–30]. However, the evidence is inconsistent, and a definitive conclusion has yet to be reached. To our knowledge, no study reports an association of hypertensive disorders during pregnancy with metformin administration. However, the meta-analyses’ results on the topic vary. Wang et al. (2021) conclude that metformin significantly reduced the risk of pregnancy-induced hypertension (RR 0.47, 95% CI 0.27 to 0.83; *p* = 0.01, *n* = 822) and had no effect on pre-eclampsia incidence [38]. Feng et al. (2017) demonstrated lower rates of gestational hypertension and pre-eclampsia (RR 0.07, 95% CI 0.49 to 0.99; *p* = 0.04, *n* = 1660) [28]. Guo et al. (2019) suggested that pregnancy-induced hypertension risk was similar (RR 0.56, 95% CI 0.30 to 1.06; *p* = 0.08, *n* = 606) and pre-eclampsia risk was lower under metformin treatment (RR 0.57, 95% CI 0.56 to 1.74; *p* < 0.001, *n* = 3402) [20].

The MiTy trial compared the addition of either metformin or placebo to insulin treatment in women with T2DM. No difference was observed in the collective incidence of gestational hypertension, worsening hypertension, and pre-eclampsia between the compared groups [31]. On the contrary, Ainuddin et al. (2015) compared metformin alone (*n* = 16), metformin plus insulin (*n* = 90), and insulin alone (*n* = 100). Gestational hypertension was significantly less frequent in the metformin-alone group and the metformin plus insulin group compared with insulin alone (6.2%, 23.3% and 36%, respectively). Pre-eclampsia incidence was comparable across all groups [9].

### Cesarean delivery

Hyperglycemia during pregnancy is a well-known risk factor for cesarean delivery [39–41]. Three meta-analyses, including GDM patients (*n* = 706, *n* = 1270, and *n* = 2611), concluded that the risk for cesarean delivery was comparable regardless of treatment modality [20, 28, 29]. However, in a recent meta-analysis, metformin administration was associated with fewer cesarean deliveries (RR 0.86, 95% CI 0.78 to 0.95; *p* < 0.001, *n* = 1693) [38].

According to the MiTy trial, cesarean section was less likely in pregnant women with T2DM who received metformin plus insulin treatment compared with placebo plus insulin (RR 0.85, 95% CI 0.73 to 0.99; *p* = 0.031) [31]. Another study including women with pre-gestational diabetes reported that treatment with metformin plus insulin resulted in fewer cesarean deliveries compared to metformin alone or insulin alone (52.2%, 81.2%, and 82%, respectively, *p* < 0.01) [9]. Table  2 summarizes the maternal outcomes of metformin use during pregnancy.

**Table 2:** Maternal outcomes of metformin use during pregnancy

Study	Type	Indication	Control	GWG	GC	PRHD	CD
Rowan et al. (MiG) [8]	RCT	GDM	Insulin	↓	↔^a^	↔	↔
Tertti et al. [26]	RCT	GDM	Insulin	↔	↑	↔	↔
Ainuddin et al. [10]	RCT	GDM	Insulin	↓	↔^b^	↔	↔
Eid et al. [27]	RCT	GDM	Insulin	↓		↔	↔
Gui et al. [29]	SR/MA	GDM	Insulin	↓	↔^a^	↓	↔
Balsells et al. [30]	SR/MA	GDM	Insulin	↓		↓	↔
Zhao et al. [25]	SR/MA	GDM	Insulin	↓	↔^a^	↓	↔
Feng et al. [28]	SR/MA	GDM	Insulin	↓	↑	↓	↔
Guo et al. [20]	SR/MA	GDM	Insulin	↓	↔	↓	↔
Ainuddin et al. [9]	RCT	T2DM	Insulin	↓	↔	↓	↔
Feig et al. (MiTy) [31]	RCT	T2DM	Placebo + insulin	↓	↑	↔	↓
Løvvik et al. (PregMet2) [24]	RCT	PCOS	Placebo	↓		↔	↔
Brand et al. [19]	Cohort	PCOS, GDM, T2DM	Insulin				↔

*CD* Cesarean delivery, *GC* Glycemic control, *GDM* Gestational diabetes mellitus, *GWG* Gestational weight gain, *Met* metformin, *PCOS* polycystic ovarian syndrome, *PRHD* Pregnancy-related hypertensive disorders, *RCT* Randomized controlled trial, *SR/MA* systematic review and meta-analysis, *T2DM* type 2 diabetes mellitus

↑ increased rates, ↓ decreased rates, ↔ no change

^a^Glucose targets were reached sooner in the metformin group

^b^HbA1c concentrations were within the normal range at 36–37 gestational weeks but slightly higher in metformin-treated patients

## Maternal outcomes: metformin for other indications

Metformin use in pregnant women with polycystic ovary syndrome (PCOS) and obesity is a flourishing research field. Both populations are at increased risk of GDM and other pregnancy-related complications, partially attributed to the hyperglycemia and insulin resistance that characterize these conditions [42]. As an insulin-sensitizing agent, metformin reverses insulin resistance and minimizes the risk of complications. In pre-gestational diabetes, the treatment protocol starts during the first trimester [9, 17, 24]. Conversely, in GDM, treatment typically begins after 24–28 gestational weeks, when universal screening is conducted. The variation in treatment initiation affects the duration of metformin exposure and could result in differences in observed outcomes, and efficacy fluctuations, with the need for additional insulin ranging from 20.9% to 84.9%, as mentioned previously [9, 26].

The PregMet2 study included 478 pregnant women previously diagnosed with PCOS; 244 were randomized to metformin and 243 to a placebo group. Treatment was initiated during the first trimester of pregnancy. Metformin did not have any protective effect over developing GDM or hypertensive disorders during pregnancy [24]. Another RCT has also demonstrated this lack of association [43]. Neither study reported any substantial adverse effect on the mother or the offspring.

The available literature has been methodically evaluated by several meta-analyses. Zeng et al. merged the findings from 13 studies and concluded that preterm birth was negatively associated with the use of metformin during pregnancy, as well as pregnancy-induced hypertension (OR 0.22, 95% CI 0.13 to 0.38; *p* < 0.001) and early pregnancy loss (OR 0.19, 95% CI 0.12 to 0.28; *p* < 0.001) [44]. Lastly, GDM risk was statistically lower in the metformin group compared to placebo (OR 0.35, 95% CI 0.14 to 0.87; p = 0.02, *n* = 1080). However, the subgroup analysis exclusively in the RCTs did not confirm the association (OR 1.23, 95% CI 0.71 to 2.12; *p* = 0.46, *n* = 348) [44].

As mentioned above, metformin use in pregnancy aims to prevent the development of GDM in high-risk populations. Despite studies supporting metformin’s protective effect, most fail to establish this association [16–18, 24, 43–48]. The incidence of GDM was similar between metformin and control groups. Two meta-analyses evaluated the incidence of GDM in obese and overweight populations. Metformin did not reduce the risk of GDM in the pooled analysis [49, 50]. Despite its insulin-sensitizing abilities, metformin failed to prove efficient in preventing GDM development.

The exploration of metformin’s use in overweight and obese women during pregnancy is not limited to GDM prevention. Above-normal BMI measurements are associated with a wide range of maternal and neonatal complications, such as GDM, gestational hypertension, pre-eclampsia, cesarean section, as well as increased neonatal birth weight and large-for-gestational age (LGA) infants [51].

The GRoW study included overweight women (BMI 25–30 kg/m2), the EMPOWaR included obese women (BMI ≥ 30 kg/m2), and the study of Syngelaki et al. included women with BMI > 35 kg/m2 [17, 18, 47]. GDM, gestational hypertension, and birth weight were comparable across all studies in metformin and placebo groups. Pre-eclampsia rate did not differ between the metformin and placebo groups in the GRoW and EMPOWaR studies, but Syngelaki et al. reported a statistically significant reduction in frequency (OR 0.24, 95% CI 0.10 to 0.61; *p* = 0.001). LGA rate was similar in the GRoW trial and Syngelaki et al., while the EMPOWaR trial did not evaluate this outcome [17, 18, 47]. Notably, in a stratified analysis of the GRoW trial according to maternal BMI, the results indicated metformin’s protective effect on macrosomia (birth weight over 4000 g) in the overweight group compared with the obese [47]. The GRoW and EMPOWaR studies found no difference in overall gestational weight gain, while Syngelaki et al. reported reduced weight gain in the metformin group. A subsequent meta-analysis of the three RCTs supported that there is insufficient evidence to establish metformin’s use during pregnancy [16]. The EudraCT trial is currently ongoing and aims to evaluate metformin administration during pregnancy in obese patients after risk stratification and detection of high-risk individuals [52].

## Neonatal outcomes

### Gestational age/preterm birth

Numerous studies support that metformin administration in GDM treatment results in earlier gestational age compared with the control group [8, 20, 29, 30]. In the MiG trials, although the results were statistically significant (38.3 ± 1.4 vs 38.5 ± 1.3, *p* = 0.02), they were not clinically significant [8]. Similarly, three meta-analyses showed a mean difference (MD) of −0.14, 0.16 and 0.23 weeks [20, 29, 30]. Balsells et al. commented that although the net effect of metformin on gestational age was small (−0.16 weeks), it coincided with a 50% elevation of preterm birth rate (RR 1.50, 95% CI 1.04 to 2.16; *p* = 0.03, *n* = 1299) [30]. Guo et al. found no significant difference in the incidence of preterm birth; however, heterogeneity between studies was significant (RR 0.90, 95% CI 0.51 to 1.58; *p* = 0,71, *I*^*2*^ = 71%, *n* = 2943) [20]. On the contrary, metformin was not correlated with gestational age or prematurity in T2DM patients [9, 31], although no meta-analyses are available for this population.

Additionally, prematurity was examined in women with PCOS, comparing metformin to placebo. PregMet and PregMet2 found similar rates of preterm births, trending towards a protective effect of metformin [24, 43]. Moreover, a meta-analysis of this population described a significantly lower prevalence of preterm births in the metformin group (OR 0.37, 95% CI 0.20 to 0.68; *p* = 0.002, *n* = 1606). The authors also reported that metformin promoted term gestation (OR 5.23, *p* < 0.001) [44].

Finally, a register-based cohort study examined the effects of metformin administration on GDM, T2DM, and PCOS. Infants of mothers who received combination treatment with metformin and insulin were more likely to be preterm compared to infants exposed exclusively to insulin (OR 1.46, 95% CI 1.10 to 1.95). Notably, the rate of preterm deliveries was similar between metformin-alone and insulin-alone groups [19].

### Congenital anomalies

The transplacental passage of metformin raises concerns about its effects on fetal growth, especially when taken during fetal organogenesis (4^th^ to 12^th^ gestational week) [53]. The current literature on the use of metformin during the first trimester is limited but reassuring regarding the risk of major congenital malformations (MCM). Studies consistently show that metformin does not increase the overall incidence of MCM compared to insulin [19–21, 53, 54].

In their cohort study, Lin et al. pointed out a lower risk of congenital malformations in women with pre-existing T2DM treated with metformin compared with those treated with insulin (OR 0.51, 95% CI 0.27 to 0.94; *p* = 0.032). The reasons are unclear, but it is tempting to speculate that women treated with metformin may have milder diabetes than those on insulin therapy [55]. A case-control study involving 50,167 babies found a higher risk of pulmonary valve atresia after metformin exposure, compared to controls, after adjustment for diabetes and other confounders. The authors recommended further surveillance to increase the sample size and follow up on the cardiac signal, as they suspect this finding to be coincidental [56].

### Birth weight/SGA/LGA/macrosomia

Neonates with a birth weight below the 10th percentile are considered small-for-gestational-age (SGA), while those above the 90th percentile are considered large-for-gestational-age (LGA). Macrosomic neonates exceed 4000 g at birth. In the meta-analyses of Guo et al. and Tarry-Adkins et al., metformin-exposed neonates weighed less, with a mean difference of 114.48 (95% CI 37.32 to 191.64; *p* < 0.01) and 107.69 (95% CI 182.70 to 32.68; *p* = 0.005), respectively. Additionally, macrosomia incidence was significantly lower, but no difference was observed in SGA and LGA infant rates [20, 57].

Regarding pre-gestational diabetes, Ainuddin et al. estimated comparable birth weight across all groups – metformin alone, insulin alone and combination treatment [9]. The MiTy study reported a statistically significant reduction in birth weight, elevation in the rate of SGA and reduction in the rate of macrosomia in offspring exposed to insulin and metformin combined, compared to insulin with placebo (*p* = 0.0016, *p* = 0.026, *p* = 0.046 respectively). The incidence of LGA was similar in both groups [31]. Birth weight was not affected by metformin treatment in the offspring of women with PCOS [24, 43]. Another cohort study reported that metformin-exposed neonates were more likely to be SGA (OR 1.65, 95% CI 1.16 to 2.34), while the combination therapy group presented an increased risk for LGA [19].

### Neonatal hypoglycemia

Various studies support that metformin for the treatment of GDM reduces the risk of neonatal hypoglycemia in the offspring [8, 10, 20, 28, 38]. The threshold point of hypoglycemia was not consistent throughout studies, ranging from glucose concentrations <1.4 to <2.6 mmol/L [8, 9]. Ainuddin et al. did not confirm metformin’s protective effect in women with T2DM. However, the combination of metformin with insulin resulted in a significant decrease in neonatal hypoglycemia (metformin plus insulin: 7.8%, insulin alone: 30%, *p* < 0.01) [9]. The MiTy trial reported a comparable incidence of neonatal hypoglycemia between insulin plus metformin versus insulin plus placebo in patients with T2DM (RR 0.82, 95% CI 0.52 to 1.30) [31]. In a retrospective cohort study, metformin was associated with a reduced risk for neonatal hypoglycemia, regardless of treatment indication. Surprisingly, the combination of metformin with insulin was associated with an increased risk for hypoglycemia in neonates [19].

### Other neonatal complications

The collective verdict from multiple RCTs and meta-analyses is that metformin administration during pregnancy is not associated with NICU admissions, respiratory distress syndrome, or reduced APGAR score at 5 min [9, 10, 17, 20, 22, 26, 29, 31, 38, 43, 47]. Table  3  summarizes the neonatal outcomes of metformin use during pregnancy.

**Table 3:** Neonatal outcomes of metformin use during pregnancy

Study	Type	Indication	Control group	GA	PB	CA	BW	MS	LGA	SGA	NH	APGAR	NICU	RDS
Rowan et al. (MiG) [8]	RCT	GDM	Insulin	↓	↑		↔				↓	↔		↔
Niromanesh et al. [75]	RCT	GDM	Insulin	↔	↔	↔	↓	↔	↓	↔	↔	↔	↔	↔
Spaulonci et al. [76]	RCT	GDM	Insulin	↔		↔		↔			↓	↔		↔
Tertti et al. [26]	RCT	GDM	Insulin	↔	↔		↔					↔	↔	
Ainuddinn et al. [10]	RCT	GDM	Insulin				↓		↔	↔	↓	↓		
Rowan et al. (MiG TOFU) [22]	RCT	GDM	Insulin	↓	↔		↔		↑	↔				
Eid et al. [27]	RCT	GDM	Insulin	↔	↔	↔	↓	↓	↓	↔	↓	↔	↔	↔
Gui et al. [29]	SR/MA	GDM	Insulin	↓	↑	↔	↔		↔	↔	↔	↔	↔	↔
Balsells et al. [30]	SR/MA	GDM	Insulin	↓	↑		↔	↔	↔	↔	↔^a^	↔	↔	↔
Feng et al. [28]	SR/MA	GDM	Insulin		↓				↔		↓			↔
Guo et al. [20]	SR/MA	GDM	Insulin	↓	↔	↔	↓	↓	↔	↔	↓	↔	↓	↔
Tarry-Adkins et al. [57]	SR/MA	GDM	Insulin				↓	↓	↓	↔				
Wang X. et al. [38]	SR/MA	GDM	Insulin	↓	↔	↔	↓	↓	↓	↔	↓	↔	↓	↔
Ara Ainuddin et al. [9]	RCT	T2DM	Insulin	↔			↔	↔		↑	↔^b^	↔	↓	
Feig et al. (MiTy) [31]	RCT	T2DM	Placebo + insulin	↔	↔	↔	↓		↔	↑	↔			↔
Lin et al. [55]	Cohort	T2DM	Insulin		↔	↓			↔	↔		↔		
Vanky et al. (PregMet) [43]	RCT	PCOS	Placebo		↔		↔					↔		
Løvvik et al. (PregMet2) [24]	RCT	PCOS	Placebo	↔	↔									
Zeng et al. [44]	SR/MA	PCOS	Placebo		↓									
Syngelaki et al. [17]	RCT	Obesity	Placebo		↔		↔		↔		↔	↔	↔	↔
Dodd et al. (GRoW) [47]	RCT	Overweight, obesity	Placebo	↔	↔		↔		↔	↔			↔	
Gilbert et al. [21]	SR/MA	PCOS, diabetes	No Met exposure			↓								
Given et al. [56]	Case-control	Diabetes, PCOS, infertility or combination	No Met exposure			↔								↓
Diav-Citrin et al. [53]	Cohort	PCOS, T2DM	Insulin and non-teratogenic exposure		↔	↔								
Scherneck et al. [54]	Cohort	PCOS/ Fertility, diabetes, insulin resistance	No Met exposure		↔	↔	↔							
Brand et al. [19]	Cohort	PCOS, GDM, T2DM	Insulin		↔	↔	↓		↔	↑	↔			

*BW* birth weight, *CA* congenital anomalies, *GA* gestational age at delivery, *GDM* gestational diabetes mellitus, *LGA* large-for-gestational age, *Met* metformin, *MS* macrosomia, *NH* neonatal hypoglycemia, *NICU* admission to neonatal intensive care unit, *PB* preterm birth, *PCOS* polycystic ovary syndrome, *RCT* randomized controlled trial, *RDS* respiratory distress syndrome, *SGA* small-for-gestational age, *SR/MA* systematic review and meta-analysis, *T2DM* type 2 diabetes mellitus

↑ significant increase, ↓ significant reduction, ↔ no significant difference

^a^Significantly lower incidence of severe neonatal hypoglycemia was found in the metformin group compared with the insulin group. Severe neonatal hypoglycemia was defined by authors or by requiring intravenous glucose or NICU admission

^b^Significantly less neonatal hypoglycemia was found in the metformin

## Childhood outcomes

Offspring of mothers with GDM from the MiG trial were divided into medical centers in Adelaide and Auckland to examine the long-term effects of intrauterine exposure to metformin. In Adelaide (metformin: *n* = 58, insulin: *n* = 51), no difference was detected in anthropometric measures at the 2- and 7-year checkpoint. Conversely, in Auckland (metformin: *n* = 45, insulin: *n* = 54), the 2-year-old metformin-exposed offspring had significantly higher measurements in BMI, chest, mid-arm, waist and hip circumference, waist-to-hip ratio, subscapular and bicep folds. At the age of 9 years, they continued to have higher weight, mid-arm and waist circumference, waist-to-height ratio, and upper arm fat mass. Additionally, metformin and insulin groups had comparable body fat mass and subcutaneous, visceral, and total abdominal mass percentages. Mothers in the metformin group had higher BMI and the associations remained after adjustment for maternal BMI and weight [22]. A follow-up study of the MiG offspring reported no neurodevelopmental effect at the age of 2 years [58]. Another meta-analysis indicated that metformin exposure resulted in heavier offspring with similar height between 18 months and 2 years old; however, the results were inconsistent at 5-9 years old. BMI was significantly higher in the metformin-exposed group of the same age (MD 0.78, 95% CI 0.23 to 1.33; *p* = 0.005) [57].

Corresponding studies were conducted on the offspring of women with PCOS, comparing metformin to placebo. Offspring exposed to placebo demonstrated higher weight, BMI, and overweight/obesity ratio at 4 years of age, with no difference in height. This relation was apparent from 6 months of age. Adjustment for pre-pregnancy maternal BMI did not alter the results [59]. Offspring from the PregMet trial were examined in the PedMet study at 5–10 years old. Data showed a link between metformin exposure and increased BMI, obesity, waist circumference, and waist-to-height ratio, with no difference in height alone. Maternal pre-pregnancy BMI > 30 kg/m^2^ was correlated with higher adiposity in the metformin group [23]. Lastly, a register-based cohort compared the long-term effects of metformin-alone, metformin plus insulin, and insulin-alone intrauterine exposure. No difference was reported in obesity and motor-social developmental outcomes [19].

Although there is limited available data, intrauterine exposure to metformin has been accused of impairing the reproductive health of the offspring. Animal studies have shown that metformin exposure *in utero* is associated with reduced testicular size and growth, as well as with reduced testosterone production [60, 61]. However, no difference was noted in the testicular size of boys aged 33 to 85 months born by mothers with GDM randomized to metformin or insulin [62].

## Pharmacokinetics

Metformin’s transplacental transmission is one major concern that impedes its official authorization in pregnancy. The concentrations in the umbilical cord blood range from 50-100% of the maternal [63]. Metformin is a substrate of many cellular transportation systems, including organic cation transporter proteins (OCT3), expressed in the placental tissue and are the main effectors in transplacental passage. OCT3 expression is low during the first trimester and increases progressively with gestational age [64]. In parallel, the mitochondria, which are metformin’s main site of action, are few and immature at the early stages of gestation and develop gradually with fetal growth [65]. Consequently, the above mechanism supports metformin’s safety in early gestation and the absence of risk for congenital anomalies, as demonstrated in clinical studies [20, 21, 54].

However, the overall effect of metformin administration in pregnancy has yet to be determined. It has been suggested that metformin can affect fetal growth, nutrient bioavailability, and epigenetic programming [64, 65]. Metformin shifts the cellular metabolism from anabolic to a catabolic state, and suppresses growth and the transplacental transmission of nutrients (amino acids, glucose) – despite the nutrient-rich environment [65]. The resulting calorie depletion could explain the observed trend for reduced neonatal birth weight and SGA incidence following metformin use in pregnancy [64]. The catabolic state and nutrient restriction sustain a developmental environment that predisposes to cardiometabolic disease in adulthood (“thrifty hypothesis”) [66].

Prenatal metformin exposure leads to significant changes in the hepatic gene expression profile. The insulin-induced gene-1 (Insig-1) is involved in lipogenesis and adipogenesis, and its expression is upregulated in adipose tissue in response to a high-fat diet. In response to prenatal metformin exposure, Insig-1 mRNA expression was upregulated approximately threefold in male mice offspring, and a similar trend was observed in females without statistical significance [67]. H19 gene expression is also upregulated. Normally, its expression is negligible in adult liver, but it increases in individuals with T2DM. H19 overexpression reduces methylation and upregulates hepatocyte nuclear factor 4α (Hnf4α) expression, activating of the gluconeogenic program, resulting in altered hepatic differentiation and premature gluconeogenesis gene expression [68]. Additionally, data suggest an implication of metformin in the bioavailability of methyl donors, such as folic acid and vitamin B12, as well as a direct effect on one-carbon metabolism. These changes result in DNA hypomethylation and differential epigenetic signaling. Thus, metformin activity during fetal growth can permanently alter the metabolic programming of the body [64].

Metformin’s effect along with the diet followed during pregnancy was examined. Specifically, metformin administration to mothers who followed a regular diet during pregnancy predisposed to offspring obesity and male-specific glucose intolerance during adulthood in response to a high-fat diet [69]. Conversely, metformin administration to mothers who followed a high-fat diet during pregnancy had a protective effect on metabolic programming, resulting in reduced weight, adipose tissue gain, and glucose intolerance in both sexes when exposed to a high-fat diet during adulthood [67]. To correctly appreciate the results of in vivo experiments on rodents, it is critical to acknowledge the differences in fetal development between mice and humans. Mice are born immediately after organogenesis and organ maturation occurs during the neonatal period. On the contrary, most organ development in humans is achieved in utero [70].

Another researched field is the mechanism of metformin’s protective effect over gestational hypertensive disorders, which has been demonstrated in multiple studies [9, 25, 28, 30]. The most prominent hypothesis attributes metformin’s protective qualities to a pro-angiogenic effect. Elevation of pro-angiogenic factors like vascular endothelial growth factor (VEGF) and matrix metalloproteionase-2 (MMP-2) and inhibition of anti-angiogenic modulators like sFlt-1 and sEng, facilitate adequate trophoblast invasion and fetoplacental circulation, thus reducing hypertensive disorders [71, 72]. A different approach focuses on a potential anti-inflammatory action, thereby reducing endothelial dysfunction. Specifically, metformin’s use resulted in a reversal of lipopolysaccharide (LPS)-induced pre-eclampsia in mice, with concomitant suppression of the nFkB pathway and inflammatory modulators. Interestingly, a reduction of CRP concentrations was noted in pregnant women taking metformin compared with placebo. However, no difference was observed when compared with insulin. The authors concluded that the anti-inflammatory effect might be attributed to a general hypoglycemic effect and not to a discrete property of metformin itself [71, 73]. Based on metformin’s speculated protective endothelial effect, its utility in preterm pre-eclampsia for prolonging gestation is being examined, without evidence yet [74].

## Conclusions

In conclusion, metformin has the potential to constitute an alternative to insulin treatment during pregnancy, as it has equally effective results on glycemic control at a significantly lower cost. Also, the oral administration route is an inherent advantage, which enhances compliance and favors metformin in patients’ preferences. Nevertheless, its use during pregnancy remains controversial. The available data support that metformin is not associated with congenital defects. Moreover, metformin administration was associated with reduced maternal weight gain, incidence of hypertensive disorders, and neonatal hypoglycemia. However, evidence indicating altered fetal programming and predisposition to childhood obesity and metabolic syndrome during adulthood impede metformin’s universal use during pregnancy. As critical questions still require a final verdict, ongoing research on the field must be conducted.
